# Is early childhood education associated with better midlife cognition, especially for children facing socioeconomic marginalization?

**DOI:** 10.1371/journal.pone.0343880

**Published:** 2026-04-15

**Authors:** Whitney Wells, Jillian Hebert, Chloe W. Eng, Catherine dP. Duarte, Anusha M. Vable

**Affiliations:** 1 Department of Epidemiology and Biostatistics, University of California San Francisco, San Francisco, California, United States of America; 2 School of Public Health, Washington University in St. Louis, St. Louis, Missouri, United States of America; 3 Department of Epidemiology and Population Health, Stanford University School of Medicine, Stanford, California, United States of America; Yale University, UNITED STATES OF AMERICA

## Abstract

**Purpose:**

Education is a strong predictor of cognitive aging, but little work has evaluated the relationship between early childhood education (ECE) specifically and cognition in midlife and beyond.

**Methods:**

Using data from the National Longitudinal Survey of Youth 1979 Cohort (n = 7,129), we examined the relationship between attending preschool, Head Start (federally-funded free ECE targeted for low-income families), or no ECE, and midlife global cognition. We defined midlife global cognition based on immediate and delayed word recall, serial 7 subtraction, and backwards counting. We used multivariable linear regression models to estimate overall associations and associations among groups more likely eligible for Head Start, and to evaluate heterogeneities by sex, race and ethnicity, and family socioeconomic status (SES).

**Results:**

Overall, preschool, but not Head Start, was associated with better midlife cognition compared to no ECE in some models. Among families more likely eligible for Head Start, we found not only that preschool (β = 0.26; 95% CI: 0.01, 0.51) was associated with better midlife cognition, but also directionally positive evidence that Head Start (β = 0.09; 95% CI: −0.08, 0.27) was associated with better midlife cognition. Associations varied; Head Start was associated with higher midlife cognition for Black and Hispanic men and people with higher family SES marginalization compared to other groups.

**Conclusions:**

This study provides initial evidence suggesting that early childhood education may be associated with better cognition more than 40 years later, especially for Black and Hispanic men and people who faced greater socioeconomic marginalization.

## Introduction

Dementia is characterized by loss of cognitive abilities that impairs daily functioning [1]. Currently affecting over seven million US adults, the prevalence of dementia is projected to triple by 2050 [1,2]. Across the US there are stark inequities in later-life cognitive health; people with low socioeconomic status (SES) have substantially higher risk of dementia and cognitive impairment and Black individuals have up to twice the dementia risk of White individuals [3–5]. Underlying disease processes for dementia begin decades prior to diagnosis; social and structural determinants of health starting in early life interact across the lifecourse to influence development of dementia and disparities in cognitive aging [6–8]. Identifying population-level interventions to improve cognitive aging and reduce development of dementia is critical.

Early Childhood Education (ECE; education before age five) has been linked to a variety of immediate and long-term benefits that are relevant for cognitive aging. These include improved reading and math skills at age five, reduced grade repetition, and increased high school graduation, which have cascading implications into adulthood for educational attainment, labor market outcomes, residential mobility, and ability to pursue health-promoting behaviors [9–16]. Benefits of ECE are attributed to the infusion of material, curricular, and social resources it provides during a sensitive developmental period in the lifecourse [17,18], which could also have direct effects on cognitive health in later life [7]. Many of the immediate and medium-term benefits of ECE (e.g., reading and math skills in childhood, improved educational attainment, labor market participation) are themselves determinants of later cognitive health. For example, childhood reading problems and high school cognitive ability are linked with worse cognitive functioning in later life [19,20], and educational attainment is linked with development of dementia and cognitive impairment [4,21,22]. ECE may represent a valuable opportunity for intervening in early life to trigger cumulative processes that diminish the risk for development of dementia. However, little work has evaluated associations between ECE and cognitive health in midlife and beyond.

Reflecting marginalizing forces at the structural level (e.g., structural racism, structural classism) through time, ECE access in the US is strongly patterned by race, childhood SES, and their intersections [23,24]. Racially minoritized children are less likely to be enrolled in ECE than White children [23]. Racially minoritized children are also more likely to experience economic marginalization than White children [24]. There are much higher rates of ECE attendance among higher-income families, and higher access to quality ECE in more affluent communities [25–27]. While private preschool programs are a common form of ECE, these are unaffordable for many families [27,28]. The largest targeted ECE program for low-income families is the Head Start program [29]. Head Start was introduced in 1965 to provide ECE to children from low-income families and currently serves nearly one million children annually [29,30]. Head Start has been linked to improvements in childhood vocabulary [31,32], achievement tests, and high school and college completion [23,33]. Head Start has also been linked to reduced chronic health conditions including depression in adolescence [34], reduced mortality, and intergenerational benefits to educational attainment for children whose mothers attended Head Start [35,36]. Availability, however, is limited; some states have spots in Head Start programs for fewer than 10% of eligible children, suggesting inequity in access to ECE persists [28,29,37,38]. Further, whether the benefits of ECE can offset the persistent trajectory-disrupting effects of structurally marginalizing forces over the lifecourse (e.g., pushout from school, residential segregation) on racially minoritized and/or low-income families in order to impact dementia inequity has not been examined [39,40]. Examining whether ECE, and particularly programs like Head Start that are designed to facilitate equity in access to ECE, may potentially disrupt structural processes propagating inequities in cognitive aging is warranted.

In the present study, we examined the association between ECE attendance (preschool, Head Start, or no ECE) at approximately ages 3–5 and cognition at approximately age 50, which is a predictor of later-life dementia risk. Given potential confounding by sociodemographic factors in the type of ECE attended, we examined models progressively adjusted for sociodemographic factors and restricted to individuals more likely eligible to attend Head Start. Building on prior work [41–43], we investigated heterogeneity in the associations by sociodemographic factors, including sex, race and ethnicity, and family SES. We hypothesized that ECE attendance would be associated with higher midlife cognition, and that this association would be stronger among children from structurally marginalized backgrounds. While ECE may represent an important potential modifiable intervention for cognitive aging and dementia, the relationship between ECE and cognition over 40 years later is challenging to study given the temporal distance between exposure and outcome. With few longitudinal datasets that have sufficient follow-up to evaluate this association, existing research is lacking – a gap this study aims to address.

## Methods

### Study sample

The National Longitudinal Survey of Youth 1979 Cohort (NLSY79) is a national sample of people living in the US born 1957–1964. Participants were interviewed annually 1979–1994, then biennially. Inclusion criteria consisted of participants who took part in the first wave of NSLY79, who were born in the US (as these participants are more likely to have attended ECE in the US) and who completed NLSY79’s cognition module; eligible sample n = 7,468. Exclusion criteria consisted of missing the exposure (n = 339). Our resulting analytic sample was n = 7,129 (95.5% of eligible sample; eFigure 1). The NLSY79 survey is sponsored and directed by the U.S. Bureau of Labor Statistics and managed by the Center for Human Resource Research at The Ohio State University. Interviews are conducted by the National Opinion Research Center at the University of Chicago [44].

### Measures

#### Exposure.

ECE exposure was defined as attendance at preschool, Head Start, or no ECE (referent). Categories were mutually exclusive and self-reported retrospectively in 1994; “*Now think back to when you were a child. To your knowledge, did you ever attend a Head Start program when you were a pre-schooler?*” (Yes/No) and “*Did you attend any type of pre-school when you were a child?*” (Yes/No).

#### Outcomes.

Our main outcome was midlife global cognitive function (referred to as ‘midlife cognition’), a predictor for later-life dementia [45]. Midlife cognition was assessed in NLSY79 at approximately age 50 via the modified Telephone Interview for Cognitive Status (TICS-M) [46–48]. The TICS-M has been previously validated against neuropsychological testing for cognitive abilities used for diagnosing dementia and predementia [45,46]. Measures included immediate word recall (i.e., from a list of 10 words, how many words can participant correctly remember; range: 0–10), delayed word recall (i.e., after answering several unrelated questions, how many of those 10 words can participant recall; range: 0–10), serial 7 subtraction (i.e., subtract 7 from 100 five times; range: 0–5), backwards counting from 20 (binary: correct on first attempt/incorrect on first attempt), and backwards counting from 86 (binary: correct on first attempt/incorrect on first attempt). We created the score for global cognition by averaging and z-scoring the scores from two subdomains: memory domain (averaged immediate and delayed word recall measures, then z-standardized) and attention domain (z-standardized three measures: serial 7, backwards counting 20, and backwards counting 86 scores, then averaged and z-standardized again) [49,50]. Higher scores represent better cognitive functioning. The memory and attention subdomain scores are secondary outcomes as they have not been as robustly validated [46]. For additional information on how cognitive testing was performed and operationalization rules unique to NLSY79 data, see Supplemental Methods in S1 Supplement.

#### Effect modifiers.

We evaluated sex, race and ethnicity, and family SES as potential effect modifiers. Sex (mainly interviewer assigned: male/ female) was operationalized as a binary variable. Race and ethnicity (interviewer assigned race: Black/ not Black; self-reported ethnicity: Hispanic/ not Hispanic) [51] was operationalized as a categorical variable: non-Hispanic Black, Hispanic any race, non-Hispanic White (more detail in Supplemental Methods in S1 Supplement). Of note, prior research estimates that the majority of participants in NLSY’s ‘non-Black, non-Hispanic’ category were White [52]. Following recommendations from this literature, we refer to this group as ‘White’ throughout while acknowledging this language is not representative of all individuals. We conceptualize race and ethnicity as social constructs reflecting processes of social stratification and racialization–driven by structural racism–leading to structural marginalization of racialized groups.^53^ As our indicator of race and ethnicity comprises both interviewer assigned and self-reported measures, it captures different elements of this racialization process, including how others perceive and respond to an individual’s race as well as an individual’s understanding of their own ethnicity [53].

We created an index summarizing markers of family SES marginalization based on prior work [54,55], comprising six variables across three domains: family financial capital (father’s occupation, family in poverty), human capital (mother and father’s education), and social capital (father’s presence in the household and whether they ever knew their father). This index is aligned with National Center for Education Statistics recommendations for family SES to include parental educational attainment, occupational status, family income, and adjustment for family composition [56]. We allocated one point for each marker of marginalization (father unemployed, family in poverty, mother did not finish 8th grade, father did not finish 8th grade, father not in household or never known) so the index ranged from 0 to 5, and then trichotomized. (See Supplemental Methods in S1 Supplement for detailed variable operationalization).

#### Confounders.

Confounders were selected *a priori* based on their hypothesized relationships with the exposure and outcomes as supported by existing research. We included the following cofounders measured in the first wave of NLSY79 (which occurred age 14–22): year of birth, birth in the South [57–59], mother and father’s nativity [60,61], family’s poverty status in 1978, mother and father’s educational attainment as of 1979 (continuous) [61–63], and recall of the following factors corresponding to age 14: rural or urban residence [64,65], mother and father’s occupational status, and mother and father’s presence in the household [60–63]. (See Supplemental Methods in S1 Supplement for detailed variable operationalization).

### Statistical analysis

#### Primary analysis.

We tabulated descriptive statistics stratified by ECE attendance, presenting pooled statistics on our imputed dataset. To display pooled summary statistics and standard errors in Table 1, we used Rubin’s rule, as described by Nahhas 2024 [66]. This approach was also used to generate sample sizes displayed in stratified graphs.

**Table 1:** Sample characteristics by early childhood education exposure.

Variable	No Preschool/No Head Start, N = 4962^1^	Head Start, N = 1025^1^	Preschool, N = 1142^1^
**Year of birth**	1960 ± 2.24	1962 ± 1.37	1960 ± 2.22
**Female sex**	2538 (51%)	540 (53%)	560 (49%)
**Race and ethnicity**			
Non-Hispanic White^2^	2931 (59%)	164 (16%)	619 (54%)
Non-Hispanic Black	1245 (25%)	710 (69%)	352 (31%)
Hispanic	786 (16%)	151 (15%)	171 (15%)
**Born in the South**	1773 (36%)	604 (59%)	526 (46%)
**Father born in US**	4714 (95%)	987 (96%)	1087 (95%)
**Mother born in US**	4712 (95%)	986 (96%)	1073 (94%)
**Poverty prior year**	1042 (21%)	412 (40%)	204 (18%)
**Rural residence**	1147 (23%)	196 (19%)	173 (15%)
**Father’s education**			
< 8th grade	812 (16%)	242 (24%)	138 (12%)
8th grade to <High School	1365 (28%)	362 (35%)	245 (21%)
Highschool	1740 (35%)	312 (30%)	347 (30%)
> Highschool	1045 (21%)	109 (11%)	412 (36%)
**Mother's education**			
< 8th grade	542 (11%)	165 (16%)	99 (9%)
8th grade to <High School	1571 (32%)	434 (42%)	238 (21%)
Highschool	2112 (43%)	329 (32%)	437 (38%)
> Highschool	738 (15%)	97 (9%)	368 (32%)
**Father’s occupation**			
Employed, unskilled	2827 (57%)	528 (51%)	445 (39%)
Employed, skilled	1097 (22%)	73 (7%)	463 (40%)
Unemployed	1038 (21%)	424 (41%)	234 (20%)
**Mother’s occupation**			
Employed, unskilled	2075 (42%)	496 (48%)	426 (37%)
Employed, skilled	473 (10%)	72 (7%)	228 (20%)
Unemployed	2414 (49%)	457 (45%)	488 (43%)
**Father not in household/not known**	832 (17%)	346 (34%)	233 (20%)
**Mother not in household/not known**	84 (2%)	19 (2%)	9 (1%)
**Family SES index**			
Lower marginalization (0)	2861 (58%)	352 (34%)	676 (59%)
Medium marginalization (1–2)	1467 (30%)	381 (37%)	336 (29%)
Higher marginalization (3+)	634 (13%)	292 (28%)	129 (11%)
**Global cognition score**	−0.023 ± 1.02	−0.324 ± 1.13	0.092 ± 1.01
**Memory subdomain score**	0.008 ± 0.99	−0.131 ± 1.04	0.147 ± 1.04
**Attention subdomain score**	−0.024 ± 1.04	−0.345 ± 1.21	0.013 ± 1.02

^1^N(%) or Mean ± SD.

^2^NLSY created a category for “Non-Black, Non-Hispanic”, which we have labelled “Non-Hispanic White” based on prior research.

Notes: Data drawn from the National Longitudinal Survey of Youth 1979. N = 7,129. Abbreviations: Socioeconomic status (SES).

To estimate the association between ECE and midlife cognition, we performed a series of multivariable linear regression models. All models included robust standard errors. In the Baseline Model we adjusted for pre-exposure, time invariant covariates (year of birth, sex, race and ethnicity, birth in the South, and mother’s and father’s nativity). In subsequent models we adjusted for family SES factors to address potential confounding [19,28,62,67]. Given these factors were measured post-exposure (see Supplemental Methods in S1 Supplement), in the Partially SES-Adjusted Model we first adjusted for mother’s and father’s education given parent education is among the most influential SES indicators and likely more stable (less likely to have a mediating pathway from the exposure) than other SES measures [68,69]. In the Fully SES-Adjusted Model we additionally adjusted for other family SES measures: rural residence, family poverty in the prior year, mother’s and father’s occupation, and mother’s and father’s presence in the household.

#### Tests for effect modification.

To further examine the role of family SES, we examined heterogeneity in the relationship by family SES using the index of family SES marginalization. We also examined heterogeneity in the effect of ECE on midlife cognition by intersectional identities of sex, race, and ethnicity based on existing evidence for heterogeneous returns to education patterned across these factors [41–43]. We present stratified estimates using rotating reference groups and evaluated statistical evidence for effect modification by including an interaction term in the main model, using the largest group as the reference. (More detail in Supplemental Methods in S1 Supplement).

#### Estimation among people more likely eligible for Head Start.

Given Head Start eligibility is based on family income, ideally we would perform estimations restricted to eligible families, but NLSY79 does not capture eligibility criteria [29]. We hypothesize people with higher family SES marginalization are more likely eligible for Head Start, therefore we additionally used the stratified estimates for this group as a proxy for restriction to Head Start-eligible families.

#### Missing data.

We performed multiple imputation to account for missing values of all covariates and outcomes. We created two hundred imputed datasets using the Multivariate Imputation by Chained Equations (MICE) package in R, and pooled estimates across imputed datasets. We created composite measures for outcomes and family SES after conducting multiple imputation. (More detail in Supplemental Methods in S1 Supplement).

#### Sensitivity analyses.

We performed several sensitivity analyses detailed in the Supplemental Methods (see S1 Supplement): [1] examining memory and attention cognition subdomains; [2] interaction by family SES using only parents’ education; [3] complete case analysis; and [4] exclusion of people born before 1960 given Head Start started in 1965.

Analysis was performed in R version 4.4.0. Code review was performed on all code, which is available on GitHub. The IRB at the University of California, San Francisco determined the research was exempt based on use of publicly available data (IRB #24–40942).

## Results

### Sample characteristics

The final sample included 1,025 (14%) people who attended Head Start, 1,142 (16%) who attended preschool, and 4,962 (70%) who attended no ECE (Table 1). People who attended Head Start were more likely to be Black, born in the South, live in poverty in 1978, have parents who didn’t finish 8th grade, a father who was unemployed, a father who did not reside in the same home as the participant, and markers of family SES marginalization. People who attended preschool were more likely to have parents with education beyond High School and in skilled employment. People who attended no ECE were more likely to be White, have lived in a rural residence, have a father in unskilled employment, have a mother who was unemployed, and less likely to be born in the South. Summary statistics and missingness in original data are in Supplemental eTable 1 and Supplemental Results in S1 Supplement.

### Association between ECE and midlife cognition

**Fig 1:**
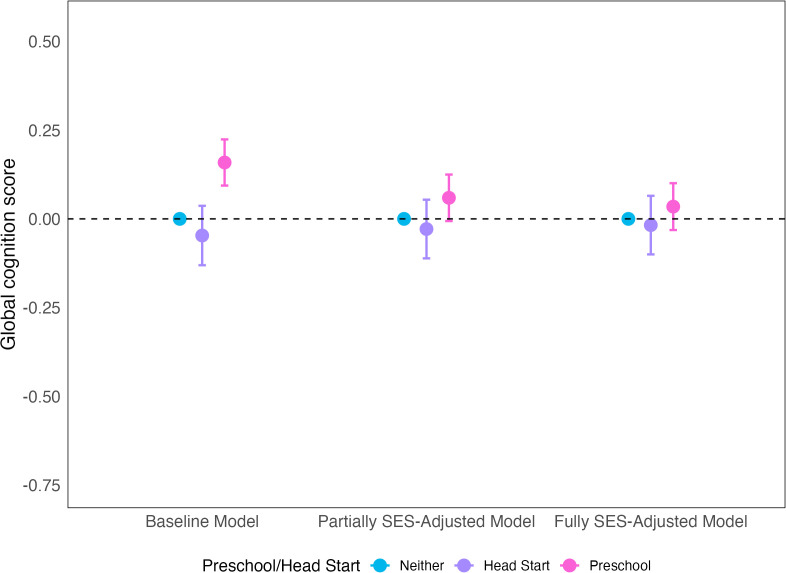
Association between exposure to early childhood education and midlife global cognition. Notes: Data drawn from the National Longitudinal Survey of Youth 1979. N = 7,129. Baseline Model adjusted for year of birth, race and ethnicity, sex, birth in a southern state, and mother’s and father’s nativity. Partially SES-Adjusted Model additionally adjusted for mother’s and father’s education as they are more likely to temporally precede the exposure (early childhood education) than other family SES measures. Fully SES-Adjusted Model additionally adjusted for rural residence, family poverty in the prior year, mother’s and father’s occupation, and mother’s and father’s presence in the household.

In the full sample, compared to no ECE, attending preschool was associated with better midlife cognition in the Baseline Model (β = 0.16; 95% CI: 0.09, 0.22) and the Partially SES-Adjusted Model although the estimate just included the null (β = 0.06; 95% CI: −0.01, 0.12). In the Fully SES-Adjusted Model, preschool was no longer associated with midlife cognition although the estimate remained positive (β = 0.03; 95% CI: −0.03, 0.10). Comparing estimates across these models is warranted given that the Baseline Model did not adjust for family SES measures that are likely confounders while the Fully SES-Adjusted Model adjusted for post-exposure variables that could have mediating pathways from the exposure. In the Partially SES-Adjusted Model we attempted to partially account for family SES confounding while avoiding concerns adjusting for post-exposure variables by only adjusting for a more stable measure of family SES (parents’ education). Compared to no ECE, attending Head Start was not associated with better midlife cognition (Baseline Model: β = −0.05; 95% CI: −0.13, 0.04; Partially SES-Adjusted Model: β = −0.03; 95% CI: −0.11, 0.05; Fully SES-Adjusted Model: β = −0.02; 95% CI: −0.10, 0.06) (Fig 1, Supplemental eTable 2 in S1 Supplement).

#### Interactions by family SES.

We found statistical evidence of effect modification for Head Start (but not preschool) by family SES marginalization. Compared to those with lower family SES marginalization, those with higher family SES marginalization showed a more positive association between Head Start and midlife cognition (interaction β = 0.19; 95% CI: −0.03, 0.40; p = 0.09; Supplemental eTable 5 in S1 Supplement).

**Fig 2:**
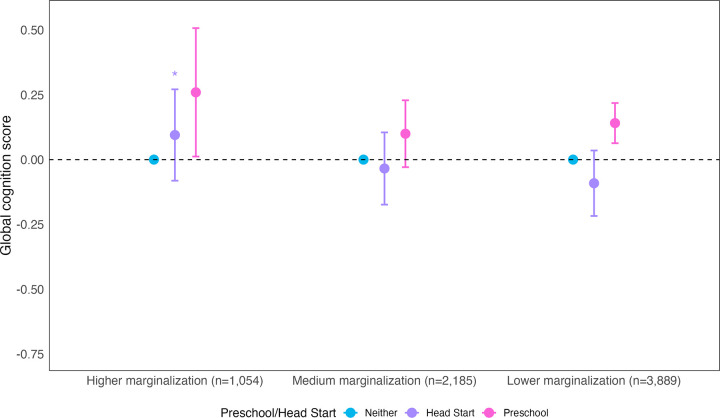
Association between exposure to early childhood education and midlife global cognition, by index of family socioeconomic status marginalization. Notes: Data drawn from the National Longitudinal Survey of Youth 1979. N = 7,129. Models adjusted for year of birth, race and ethnicity, sex, birth in a southern state, and mother’s and father’s nativity. Coefficients were obtained using rotating reference groups. Asterisks denote statistical significance for the interaction terms, which are presented in Supplemental eTable 5 in S1 Supplement. Interaction terms represent the additional difference in midlife cognition for Head Start (vs no ECE) or preschool (vs no ECE) for the given subgroup compared to the reference group of lower marginalization. *Interaction term<0.1.

In stratified results, among people more likely eligible for Head Start (with higher family SES marginalization), compared to no ECE, both preschool (β = 0.26, 95% CI: 0.01, 0.51) and Head Start (β = 0.09, 95% CI: −0.08, 0.27) were associated with better midlife cognition, although the estimate for Head Start included the null. Among those with lower family SES marginalization, preschool was associated with better midlife cognition (β = 0.14, 95% CI: 0.06, 0.23) and Head Start with worse midlife cognition (β = −0.09, 95% CI: −0.22, 0.04), although the estimate for Head Start included the null. (Full results, including medium marginalization, in Fig 2, Supplemental eTable 3 in S1 Supplement).

#### Interactions by sex by race and ethnicity.

We also found statistical evidence of effect modification for Head Start (but not preschool) by sex and by race and ethnicity. Compared to White women, there was a more positive association between Head Start and midlife cognition for Black men (interaction β = 0.31; 95% CI: 0.04, 0.57; p = 0.02) and Hispanic men (interaction β = 0.31; 95% CI: 0.00, 0.62; p = 0.05) and a more negative association between Head Start and midlife cognition for White men (interaction β = −0.29; 95% CI: −0.62, 0.05; p = 0.09) (Supplemental eTable 6 in S1 Supplement).

**Fig 3:**
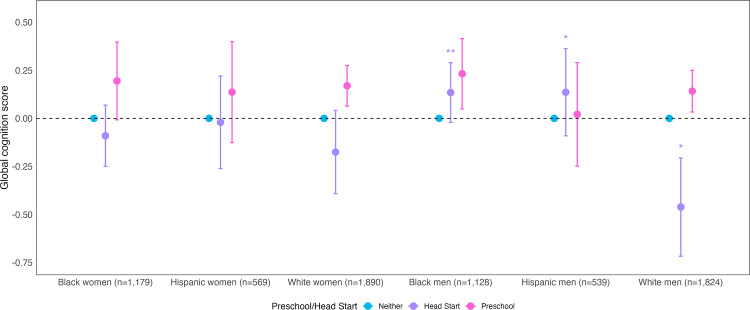
Association between exposure to early childhood education and midlife global cognition, by sex, race, and ethnicity. Notes: Data drawn from the National Longitudinal Survey of Youth 1979. N = 7,129. Models adjusted for year of birth, birth in a southern state, and mother’s and father’s nativity. Coefficients were obtained using rotating reference groups. Asterisks denote statistical significance for the interaction terms, which are presented in Supplemental eTable 6 in S1Supplement. The interaction terms represent the additional difference in midlife cognition for Head Start (vs no ECE) or preschool (vs no ECE) for the given subgroup compared to the reference group of White women. *Interaction term <0.1, **Interaction term <0.05. NLSY created a category for “Non-Black, Non-Hispanic”, which we have labelled “Non-Hispanic White” based on prior research.

In stratified results, among Black men, preschool (β = 0.23; 95% CI: 0.05, 0.42) and Head Start (β = 0.13; 95% CI: −0.02, 0.29) were associated with better midlife cognition, although the estimate for Head Start included the null. Among Hispanic men, preschool was not associated with better midlife cognition (β = 0.02; 95% CI: −0.25, 0.29) and Head Start was associated with better midlife cognition (β = 0.14; 95% CI: −0.09, 0.36), although the estimate for Head Start included the null. Among White women, preschool was associated with better midlife cognition (β = 0.17; 95% CI: 0.06, 0.28) and Head Start with worse midlife cognition (β = −0.18; 95% CI: −0.39, 0.04), although the estimate for Head Start included the null. Among White men, preschool was associated with better midlife cognition (β = 0.14; 95% CI: 0.03, 0.25) and Head Start with worse midlife cognition (β = −0.46; 95% CI: −0.72, −0.21). (Full results, including other subgroups, in Fig 3, Supplemental eTable 4 in S1 Supplement).

### Sensitivity analyses

Results were largely substantively similar (significance varied, but point estimates largely in same direction and conclusions unchanged) when examining: [1] memory and attention cognition subdomains (Supplemental eFigure 3 in S1 Supplement); [2] interaction by family SES using only parents’ education (Supplemental eFigure 4 in S1 Supplement); [3] complete case analysis (Supplemental eFigure 5 in S1 Supplement); and [4] exclusion of people born before 1960 (Supplemental eFigure 6 in S1 Supplement). Results for the associations among people with higher family SES marginalization varied for complete case analysis and exclusion of people born before 1960, likely given this is a relatively smaller group (See Supplemental Results in S1 Supplement).

## Discussion

In a national dataset of US residents followed prospectively since adolescence, we estimated associations between ECE attendance and midlife cognition to explore the potential for ECE to serve as a population-level strategy for reducing dementia burden or inequities. We found that in the overall sample, compared to no ECE, attending preschool was associated with better midlife cognition in some models while Head Start was not associated with better midlife cognition. In estimates among people more likely eligible for Head Start, preschool remained associated with better midlife cognition, and we found evidence that suggests Head Start was associated with better midlife cognition. We observed heterogeneities in these associations; we found evidence that suggests Head Start was associated with better cognition among Black and Hispanic men and people with higher family SES marginalization, and worse cognition among White men and women and people with lower family SES marginalization.

Our finding that attending preschool was associated with better midlife cognition is consistent with research finding benefits of ECE on outcomes that may influence midlife cognition (e.g., school progress, educational attainment, and labor market outcomes into adulthood) [4,9–16,21,22,70,71]. It is worth noting that we lack detail on the quality and type of preschool experiences in our sample. While we were able to control for some factors that may influence ECE quality (such as urban/rural geography) [72], it would be valuable to adjust for measures of ECE quality as some research indicates the impact of preschool varies widely based on quality of the program [73].

Our finding that attending Head Start is not associated with better midlife cognition in the full sample but was potentially associated with better midlife cognition among those more likely eligible could be due to a few mechanisms. First, attendance at Head Start likely acts as an indicator of structural marginalization in our overall sample. Given eligibility requirements, there are sociodemographic differences in those who do and do not attend Head Start. Head Start attendance is more common among children whose families have lower income or receive welfare, as well as children in rural communities, racially minoritized children, and children of immigrants [61]. Additionally, our comparison group of no ECE likely represents a wide variety of caregiving arrangements (e.g., parental care, other relative care, non-relative care) that may vary by economic marginalization. We are limited by childhood sociodemographic factors in NLSY79 and likely face residual confounding in estimates in the overall sample. Our results restricted to individuals more likely eligible for Head Start help ensure the sample attending preschool or no ECE is more similar to the sample attending Head Start on sociodemographic factors and likely provides the most accurate estimate for the association between Head Start and midlife cognition. However, our index of family SES marginalization does not precisely capture Head Start eligibility, therefore examining estimates stratified across all levels of this index also provides context for the pooled results. In our overall sample, those without markers of family SES marginalization make up a large proportion of the sample and represent a heterogeneous group. Additionally, among White men, Head Start (but not preschool) was associated with worse midlife cognition compared to attending no ECE; this could similarly reflect that White men who did not attend ECE may have been a relatively more advantaged group compared to White men who attended Head Start.

Second, children who attend Head Start are more likely to subsequently attend schools with lower funding and lower test scores [74–76]. Structural differences–shaped by capitalism, racism, and classism–in future education trajectories could undermine initial potential gains from Head Start when looking at the overall population [39].

Pooled estimates may also mask differential effects of ECE across populations. We found the association between Head Start and midlife cognition was more positive for Black and Hispanic men and people with greater family SES marginalization. Our results appear consistent with evidence of greater short- and medium-term benefits from ECE for structurally marginalized children from families with lower income, lower education, less resourced family environments, children of immigrants, and Black and Hispanic children [11,12,77–79]. Prior research has highlighted that ECE may be especially valuable for structurally marginalized children who have lower access to other enriching environments compared to structurally privileged children [71,80,81]. Our findings also appear consistent with resource substitution theory, which suggests that individuals who are more socially marginalized and have lower access to flexible resources due to structural discrimination, including structural racism and classism, will benefit more from increased access to resources such as those provided in educational spaces [82].

This study has highlighted important areas for future research. ECE settings vary widely, and future research should examine how the results differ based on the type of ECE (e.g., public vs. private; home-based vs. center based, etc.) or quality of the program. This could include adjusting for or examining heterogeneity by quality benchmarks such as child-to-staff ratio, classroom size, and training requirements for teachers. Similarly, it would be valuable for future research to account for the wide variety of caregiving arrangements that may be represented among children who do not attend ECE. To better understand the mechanisms for the relationship between Head Start attendance and later-life outcomes, research exploring potential mediating factors, such as the quality of schools subsequently attended by children who do and do not attend Head Start would be useful. Finally, future research is needed that can restrict analyses to participants known to have been Head Start eligible.

This study has several strengths. Using a longitudinal national survey capturing both ECE attendance and midlife outcomes over 40 years later provided a unique opportunity to examine more distal outcomes of health and aging than is often possible with ECE. NLSY79 is a diverse, national sample of US residents including representation across geography and by urbanicity [83]. We were able to adjust for multiple sociodemographic factors. NLSY79’s cognitive assessment module leverages validated midlife cognition measures. Finally, we were able to provide preliminary exploration of how ECE may influence inequities in cognitive aging by examining heterogeneity by sociodemographic factors.

This study also has limitations. Our ECE exposure was self-reported years after attendance, which could result in recall bias. It is possible this recall bias could be dependent or differential with respect to other factors associated with the outcome (therefore measurement bias due to unmeasured covariates is possible), but likely not directly to the outcome given the exposure was measured decades prior to expected onset of cognitive decline. Our measure of race and ethnicity collapsed all ‘non-Black, non-Hispanic’ participants into a single group, the majority of whom were White. As such, we were unable to estimate associations for American Indian or Alaska Native, Asian, and Native Hawaiian or Other Pacific Islander participants. Future research should replicate these analyses in a sample representative across racial and ethnic identities with indicators that better measure those identities. We faced limitations in temporal ordering; NLSY79 did not capture family SES prior to ECE, so it is possible that our SES adjusted models are biased due to conditioning on a mediator. Family SES was self-reported (although adolescent and maternal reports show high agreement) [84]. We lacked measures for Head Start eligibility, preventing precise restriction to eligible children. We likely have residual confounding related to unmeasured sociodemographic factors. Given limited ECE data, we may lack consistency in the exposure; [85], e.g., varying quality or duration for ‘preschool’ or ‘Head Start’, and varying care settings for ‘no ECE’ [28,86]. Missingness may be informative, although we addressed this via multiple imputation and comparison to complete case analysis. We did not include survey weighting given weighting is less appropriate for estimating causal effects rather than population-level estimates, and given we adjusted for variables used in the sampling approach [87,88]. We could also face bias from survey non-response and loss-to-follow-up.

In conclusion, this study provides preliminary evidence suggesting that ECE may be associated with better cognition over 40 years later, especially among people facing greater structural marginalization. If causal, investing in ECE may be a valuable population-level strategy for reducing dementia, and the Head Start program may help address existing disparities in dementia. Our results highlight the need for more research on the relationship between ECE and later-life health and the importance of capturing experiences with ECE in data sources studying later-life health. Our results are largely consistent with existing research on ECE and shorter-term outcomes, suggesting that expanding access to ECE for populations facing greater structural marginalization such as low-income communities may improve children’s long-term cognitive health and help reduce socioeconomic inequities in cognitive aging. The burden of dementia is projected to rise substantially, therefore identifying potential modifiable risk factors at a population-level is critical. This study is one of the first to examine ECE and midlife cognition; future research is needed to build on the hypotheses generated in this study, investigate causal pathways, and evaluate whether results generalize to other populations.

## Supporting information

S1 Supplement NLSY ECE Cognition Supplement_Clean. (DOCX)
