# Supplementary material for: Is early childhood education associated with better midlife cognition, especially for children facing socioeconomic marginalization?
Source: PLoS One. 2026 Apr 15;21(4):e0343880. doi: 10.1371/journal.pone.0343880 (PMC13082659; doi:10.1371/journal.pone.0343880)
Supplement: S1 Supplement — (DOCX) [file pone.0343880.s001.docx]

**S1. SUPPLEMENT**

**Is early childhood education associated with better midlife cognition, especially for children facing socioeconomic marginalization?**

Whitney Wells, Jillian Hebert, Chloe W. Eng, Catherine dP Duarte, Anusha M. Vable

**Table of contents:**

**Supplemental Methods and Results**

**eFigure 1.** Sample inclusion

**eFigure 2.** Conceptual framework for the relationship between early childhood education and midlife cognition, reflecting study covariates

**eFigure 3.** Association between exposure to early childhood education and midlife cognition, memory and attention subdomains

**eFigure 4.** Association between exposure to early childhood education and midlife global cognition, by parent education

**eFigure 5.** Manuscript figures, using complete case analysis

**eFigure 6.** Manuscript figures, excluding people born before 1960

**eTable 1.** Sample characteristics by early childhood education exposure, showing missingness on each variable

**eTable 2.** Detailed results: Association between exposure to early childhood education and midlife global cognition

**eTable 3.** Detailed results: Association between exposure to early childhood education and midlife global cognition, by index of family socioeconomic status marginalization

**eTable 4.** Detailed results: Association between exposure to early childhood education and midlife global cognition, by sex, race, and ethnicity

**eTable 5.** Detailed results: Association between exposure to early childhood education and midlife global cognition, with interaction term for index of family socioeconomic status marginalization

**eTable 6.** Detailed results: Association between exposure to early childhood education and midlife global cognition, with interaction term for sex, race, and ethnicity

**Supplemental Methods**

**Measures**

Outcomes

Cognitive testing in NLSY79 is performed when participants are approximately age 50.^1^ Immediate word recall is measured by showing the participant a list of 10 words and counting how many words they can correctly remember. After answering several unrelated questions, delayed word recall is then measured by counting how many words the participant can recall after this delay.^2^ NLSY79 mistakenly administered immediate word recall twice to three participants (one in 2006, two in 2008); their higher score was retained. In 2008, a survey skip pattern error caused 2,256 respondents to not be administered delayed word recall.^2^ Although this outcome therefore has high missingness, the missingness is very likely to be ‘missing at random,’ making multiple imputation appropriate for addressing the missingness. Serial 7 subtraction is measured by asking participants to subtract 7 from 100 five times (i.e. 93, 86, 79, 72, 65). Participants score 1 point for each number of correct subtractions (up to 5 points), where an error in an initial subtraction does not affect their score in later subtractions.^2^ Backwards counting is measured in two ways; quickly counting backwards from 20, and quickly counting backwards from 86.^2^ For each measurement, in line with prior work, we dichotomized the scores as 0 if the participant was incorrect on the first attempt, and 1 if they were correct on the first attempt.^3^

Covariates

NLSY79 captured race and ethnicity based on a combination of interviewer observation and self-report; interviewers were instructed to code race by observation and then inquire about ethnicity of household members age 14 and above. NLSY created a three-level coding of race and ethnicity as follows: Hispanic, Black, and Non-Black, Non-Hispanic; full detail is available in the following citation.^4^

Birth in the south was defined by NLSY79 based on state of birth (southern states included Alabama, Arkansas, Delaware, District of Columbia, Florida, Georgia, Kentucky, Louisiana, Maryland, Mississippi, North Carolina, Oklahoma, South Carolina, Tennessee, Texas, Virginia, West Virginia).^5^ Mother and father’s nativity was self-reported by the participant as: In the US, Other country, or Never knew parent. Family poverty status in 1978 was calculated by NLSY79 based on the reported family income, number of family members in the household, and national poverty income guidelines in 1978.^6^ Mother and father’s educational attainment was self-reported by the participant in continuous years. Rural or urban residence at age 14 was self-reported by the participant as: In town or city, In country-not farm, On farm or ranch; we coded the first as ‘urban’ and the latter two as ‘rural’. We coded mother and father’s occupational status when the participant was 14 as skilled, unskilled, or unemployed. Skilled/unskilled occupation was based on parental occupation self-reported by the participant; NLSY79 provided categories based on the 1970 census 3-digit occupation codes.^7^ We coded codes <300 as skilled, and codes >=300 as unskilled. Parents’ employment status was self-reported based on a question of whether the parent worked for pay (Yes/No). Mother and father’s presence in the household was defined based on two questions; whether they reported never knowing their parent when asked about parents’ nativity, and self-report of who they lived with at age 14. Who they lived with at age 14 was captured in NLSY79 with one category for ‘adult man’ and one for ‘adult woman’; e.g. categories for ‘adult man’ included father, stepfather, man relative, other man, no man, other arrangement, on my own, missing man. The last four were coded as ‘father not present in household’.

In NLSY79, several of the parent measures included valid skips if the respondent either never knew their parent or their parent was not present in the household at age 14. We coded these values as missing and imputed the values, and additionally included separate indicators for the mother and father being absent from the household or never known. We included these variables for imputation given inclusion of variables that contribute to variable missingness is important for addressing criteria of the ‘missing at random’ assumption that assumes data missingness is random conditional upon observed and included variables.^8^

In our index of family socioeconomic status (SES) marginalization, we included parent education based on mother or father not finishing 8th grade based on precedent in prior literature and as a marker of potential SES marginalization.^9,10^ We included father, but not mother’s occupation as potentially influencing childhood SES in line with prior research on childhood SES and based on demographic patterns during this period with relatively less common maternal employment and limited evidence of a relationship between maternal employment and children’s achievement.^11,12^ We did not include mother present or absent in the household as less than 2% of mothers were absent. For coding the index, the possible scores ranged from 0-5; we coded 1 point for each parent who did not finish 8th grade, 1 point for father being unemployed, 1 point if the family was reported as being in poverty, and 1 point if they never knew their father or their father was not present in the household at age 14 (more detail on each of these variables above).

**Statistical analysis**

Adjustment for family SES

We adjusted for family SES indicators given family SES is strongly associated with both ECE attendance and later cognition so likely confounds our estimate.^13–16^ However, NLSY79 captures family SES measures ten or more years after our exposure (age 14-22). As shown in eFigure 2, these measures can represent proxies for unmeasured family SES prior to the exposure, but there could be a mediating pathway from child’s ECE attendance to later family SES (e.g. parent employment). We therefore controlled for these measures in only the Partially SES-Adjusted Model and the Fully SES-Adjusted Model, as proxies for early childhood family SES. The Partially SES-Adjusted Model included only mother and father’s educational attainment given parent education is among the most influential SES indicators and may be more stable than other SES indicators (less likely to have a mediating pathway from the exposure).^17,18^

Tests for effect modification

Likelihood ratio test statistics for overall interaction terms were obtained by comparing the nested models with and without the interaction term using the D3 package in R, which uses coefficients from each imputed dataset to calculate the likelihood.

Missing data

We excluded overlapping variables from the predictor matrix (reporting never knowing a parent and reporting a parent being absent at age 14). Given missingness on parents’ occupation was informed by parents’ absence from the household, a high proportion of missing values on parents’ occupation were imputed to unemployed, which influenced the distribution of parents’ occupation following imputation (Table 1, eTable 1).

Sensitivity analyses

For secondary outcomes, we examined the relationship between ECE and midlife memory and attention cognition subdomains. We additionally assessed interaction by family SES using only mother’s and father’s educational attainment given these measures are likely more stable than other family SES measures. To examine the robustness of estimates to our approach to missing data we conducted complete case analysis. We examined estimates excluding people born before 1960 given Head Start was launched in 1965, although this reduced statistical power. We examined estimates fully interacting the effect modifier with all covariates (akin to fully stratified results) given our use of rotating reference groups due to imputed effect modifiers.

For our sensitivity analysis using complete case analysis, for parent measures with valid skips related to parent absence, we coded a separate category for parent absence to avoid excluding all participants with missing parents from the complete case analysis.

For complete case examination of potential heterogeneity by family SES, individuals with missing data on any individual components of the index of SES marginalization were not considered to score a point on that component.

**Supplemental Results**

**Sample Missingness**

Missingness by variable prior to imputation is displayed in eTable 1. For covariates, we observed the highest missingness for father’s occupation, family poverty status in the prior year, and father’s educational attainment. For outcomes, missingness was largely driven by missing data in the memory subdomain score (discussed in “Outcomes” above).

**Association between ECE and midlife memory and attention subdomains**

The association between Head Start and the memory subdomain score was positive in direction, although all estimates included the null (eFigure 3). Preschool was associated with higher memory, although this was attenuated by adjusting for family SES factors.

Head Start was associated with lower attention subdomain score, although estimates included the null after adjusting for family SES factors. Preschool was associated with higher attention in the Baseline Model, but this was null or negative in direction (estimate including the null) after adjusting for family SES factors.

**Additional sensitivity analyses**

While we did not find evidence of effect modification by parent educational attainment, in stratified analysis we observed that people with parents with lower educational attainment showed more positive associations between Head Start and midlife cognition, however estimates included the null (eFigure 4).

In complete case analysis, estimates were consistent in direction with our main analysis, with the exception of results for people with higher family SES marginalization, which represented a small group in the complete case analyses with imprecise estimates. The results from analysis using multiple imputation are likely to be a less biased estimate overall, and given that the complete case analysis disproportionately excludes more marginalized people (including those with higher family SES marginalization and Black men), this difference in sample could explain the discrepancy in results between complete case analysis and multiple imputation.

In analyses excluding people born before 1960, results were consistent in direction with reduced statistical power, with the exception of results for people with higher family SES marginalization, which no longer showed an association with Head Start or preschool (eFigure 6).

**Supplemental References**

1. Cooksey EC. Using the National Longitudinal Surveys of Youth (NLSY) to conduct life course analyses. Handbook of life course health development. 2018;561–77.

2. Topical Guide to the Data: Health. National Longitudinal Surveys. Available from: https://www.nlsinfo.org/content/cohorts/nlsy79/topical-guide/health

3. Langa KM. Langa-Weir classification of cognitive function (1995 Onward). Survey Research Center Institute for Social Research, University of Michigan. 2020.

4. Race, Ethnicity & Immigration Data. National Longitudinal Surveys: A program of the US Bureau of Labor Statistics. Available from: https://nlsinfo.org/content/cohorts/nlsy79/topical-guide/household/race-ethnicity-immigration-data

5. NLSY79 Attachment 100: Geographic Regions. National Longitudinal Survey of Youth 1979.

6. NLSY79 Appendix 2: Total Net Family Income and Poverty Data. National Longitudinal Survey of Youth 1979.

7. NLSY79 Attachment 3: Industrial and Occupational Classification Codes. National Longitudinal Survey of Youth 1979.

8. Donders ART, Van Der Heijden GJ, Stijnen T, Moons KG. A gentle introduction to imputation of missing values. Journal of clinical epidemiology. 2006;59(10):1087–91.

9. Glymour MM, Avendaño M, Haas S, Berkman LF. Lifecourse social conditions and racial disparities in incidence of first stroke. Annals of epidemiology. 2008;18(12):904–12.

10. Vable AM, Gilsanz P, Nguyen TT, Kawachi I, Glymour MM. Validation of a theoretically motivated approach to measuring childhood socioeconomic circumstances in the Health and Retirement Study. PloS one. 2017;12(10):e0185898.

11. Goldin C. The quiet revolution that transformed women’s employment, education, and family. American economic review. 2006;96(2):1–21.

12. Goldberg WA, Prause J, Lucas-Thompson R, Himsel A. Maternal employment and children’s achievement in context: a meta-analysis of four decades of research. Psychological bulletin. 2008;134(1):77.

13. Chaudry A, Morrissey T, Weiland C, Yoshikawa H. Cradle to kindergarten: A new plan to combat inequality. Russell Sage Foundation; 2021.

14. Kaplan GA, Turrell G, Lynch JW, Everson SA, Helkala EL, Salonen JT. Childhood socioeconomic position and cognitive function in adulthood. International journal of epidemiology. 2001;30(2):256–63.

15. Richards M, Wadsworth M. Long term effects of early adversity on cognitive function. Archives of disease in childhood. 2004;89(10):922–7.

16. Zhang Z, Liu H, Choi S won. Early-life socioeconomic status, adolescent cognitive ability, and cognition in late midlife: Evidence from the Wisconsin Longitudinal Study. Social Science & Medicine. 2020;244:112575.

17. Davis-Kean PE, Tighe LA, Waters NE. The role of parent educational attainment in parenting and children’s development. Current Directions in Psychological Science. 2021;30(2):186–92.

18. Reardon SF. The widening academic achievement gap between the rich and the poor. In: Social stratification. Routledge; 2018. p. 536–50.

**Supplemental Tables and Figures**


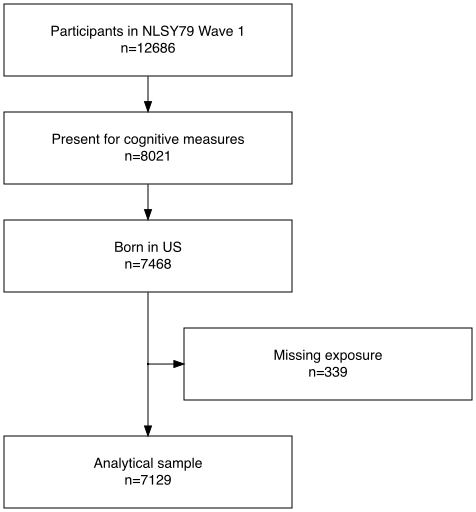


**eFigure 1.** Sample inclusion


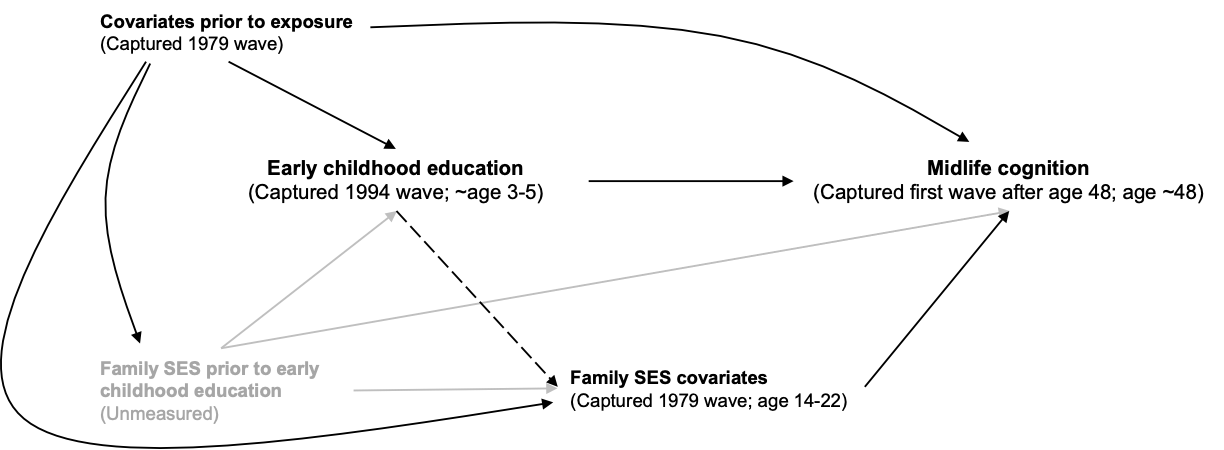


**eFigure 2.** Conceptual framework for the relationship between early childhood education and midlife cognition, reflecting study covariates

Notes: Family SES covariates may represent confounders and/or effect modifiers in the relationship between early childhood education and midlife cognition, however are only measured at age 14-22. Therefore, family SES measures prior to early childhood education are shown in gray as they are unmeasured. Family SES measures at age 14-22 are used as a proxy for these unmeasured variables. The dotted line represents a potential pathway from the exposure to measured family SES, which could lead to a partially mediating pathway for these covariates. Individual covariates included within ‘covariates prior to exposure’ and ‘family SES covariates’ are listed in the methods section. Abbreviations: Socioeconomic status (SES).


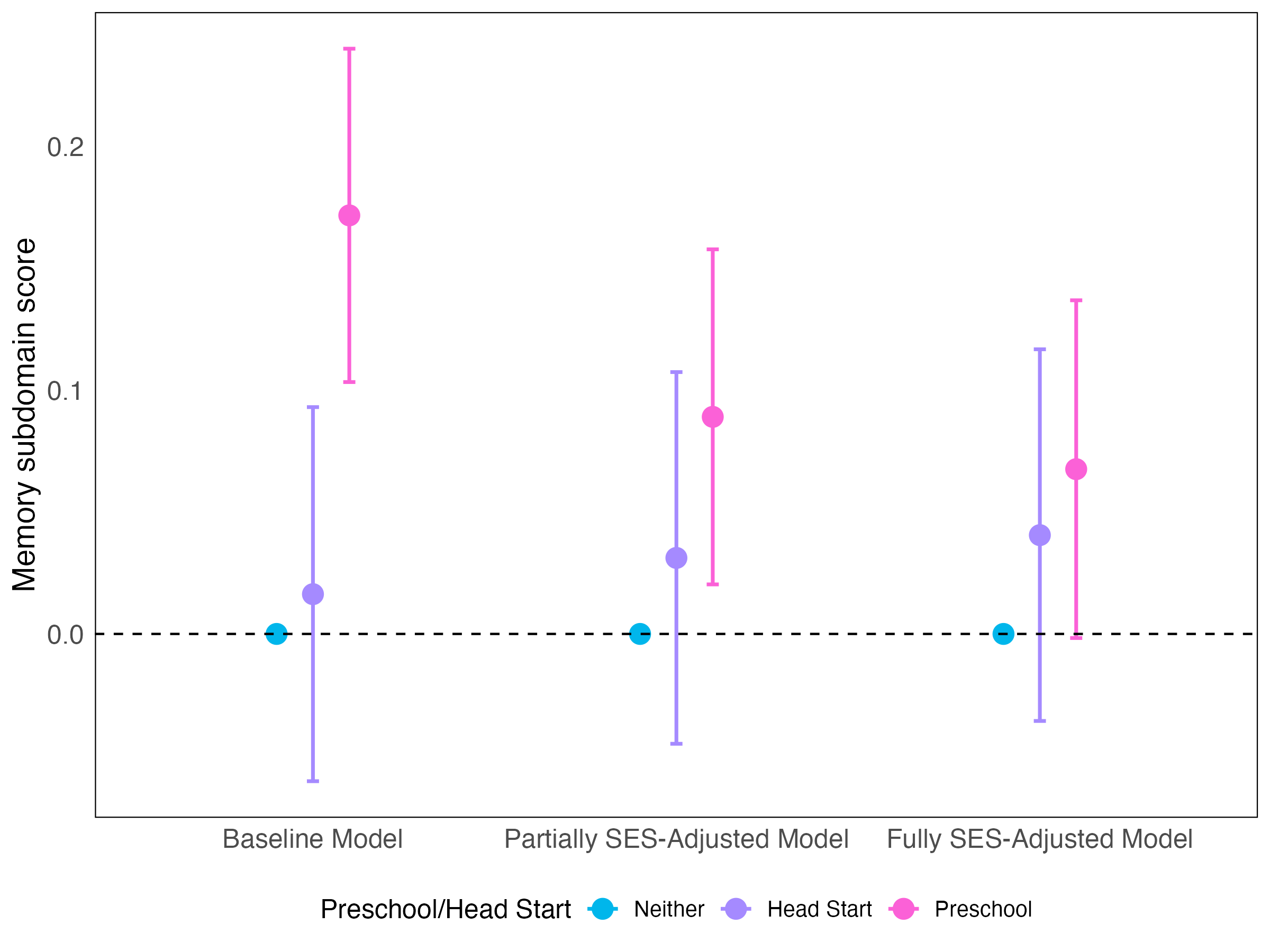


**
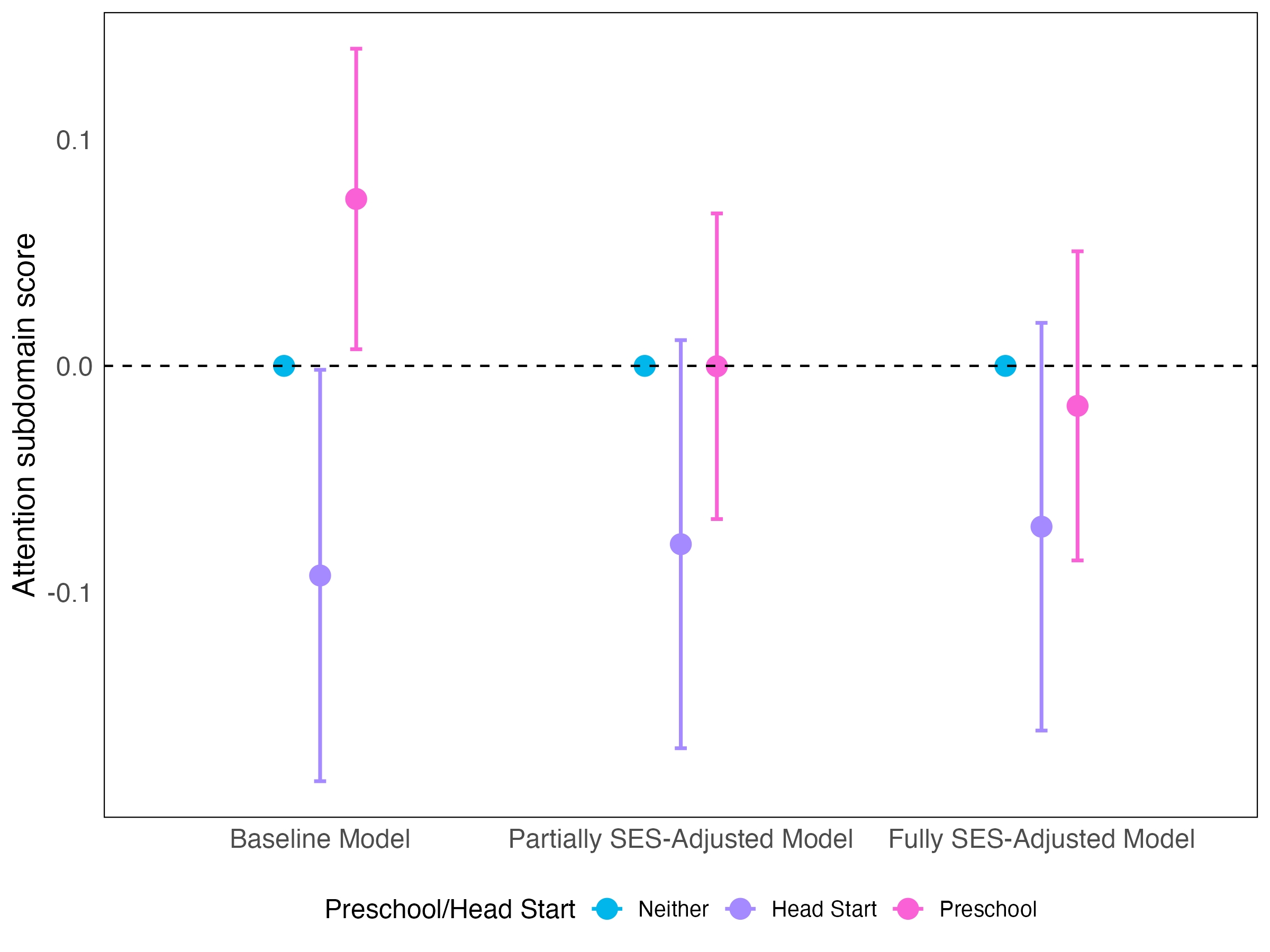
**

**eFigure 3.** Association between exposure to early childhood education and midlife cognition, memory and attention subdomains

Notes: Data drawn from the National Longitudinal Survey of Youth 1979. N=7,129. Baseline Model adjusted for year of birth, race and ethnicity, sex, birth in a southern state, and mother’s and father’s nativity. Partially SES-Adjusted Model additionally adjusted for mother’s and father’s education. Fully SES-Adjusted Model additionally adjusted for rural residence, family poverty in the prior year, mother’s and father’s occupation, and mother’s and father’s presence in the household. Subdomain cognition scores should be interpreted with caution as they have not been as robustly validated as the global cognition score.


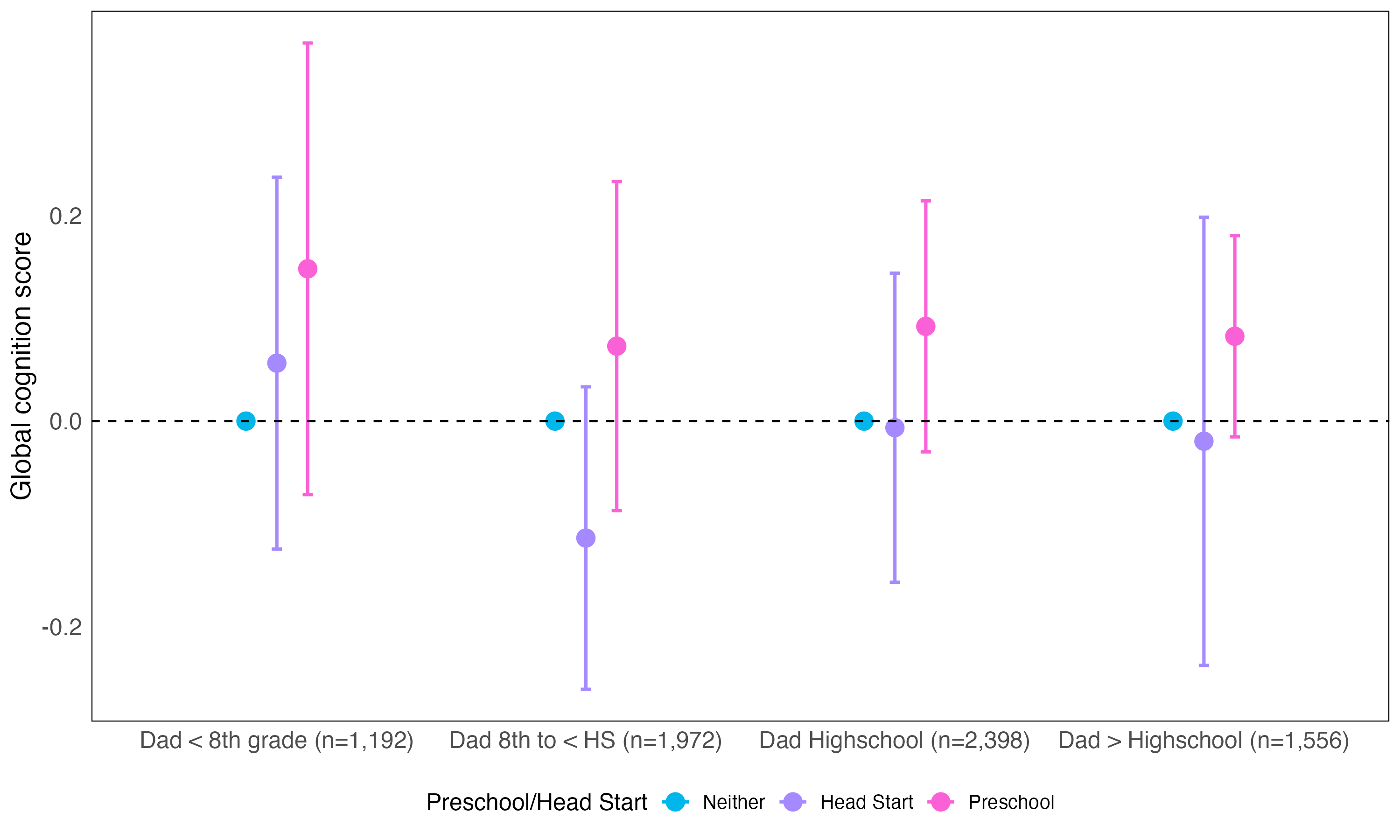

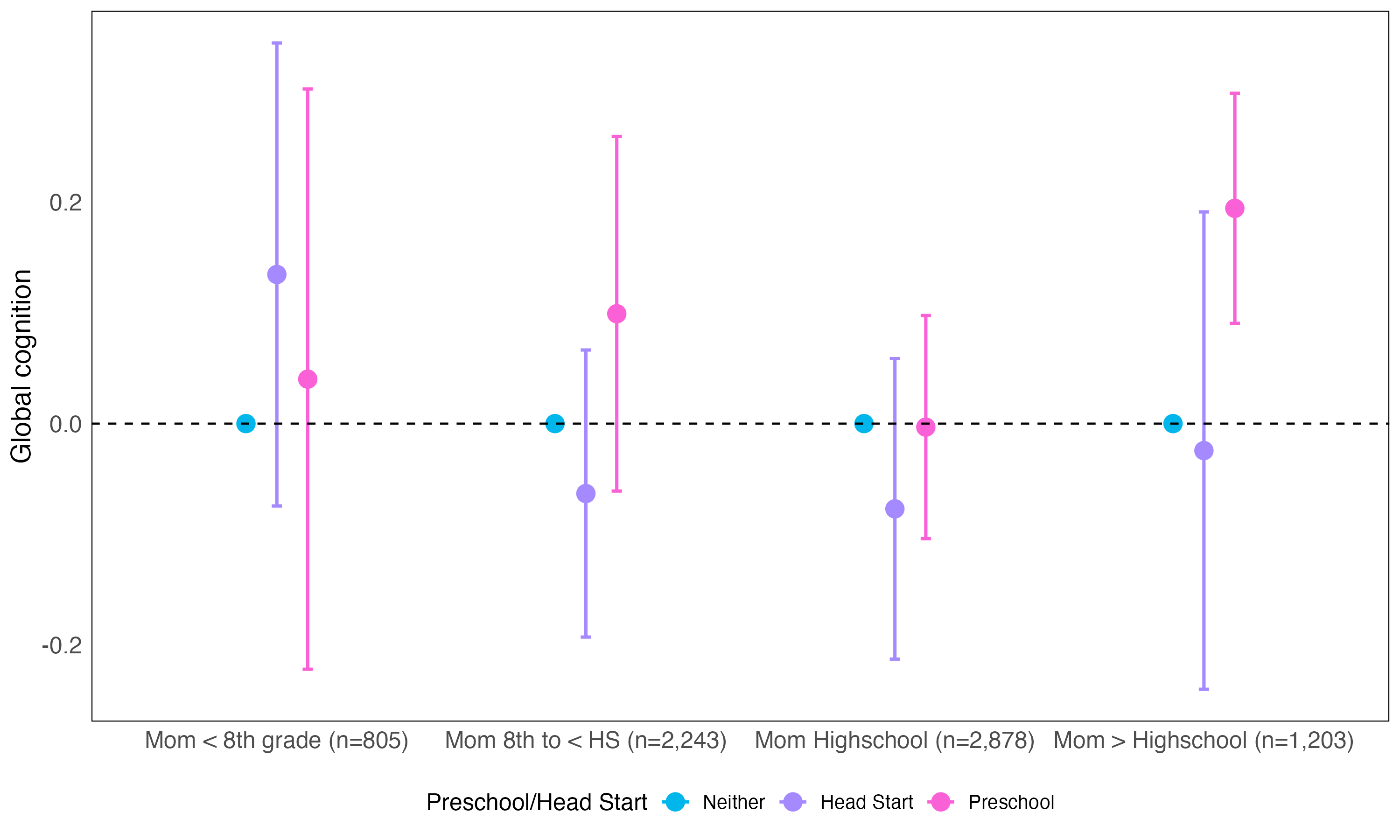


**eFigure 4.** Association between exposure to early childhood education and midlife global cognition, by parent education

Notes: Data drawn from the National Longitudinal Survey of Youth 1979. N=7,129. Models adjusted for year of birth, race and ethnicity, sex, birth in a southern state, and mother’s and father’s nativity. Coefficients were obtained using rotating reference groups.

**
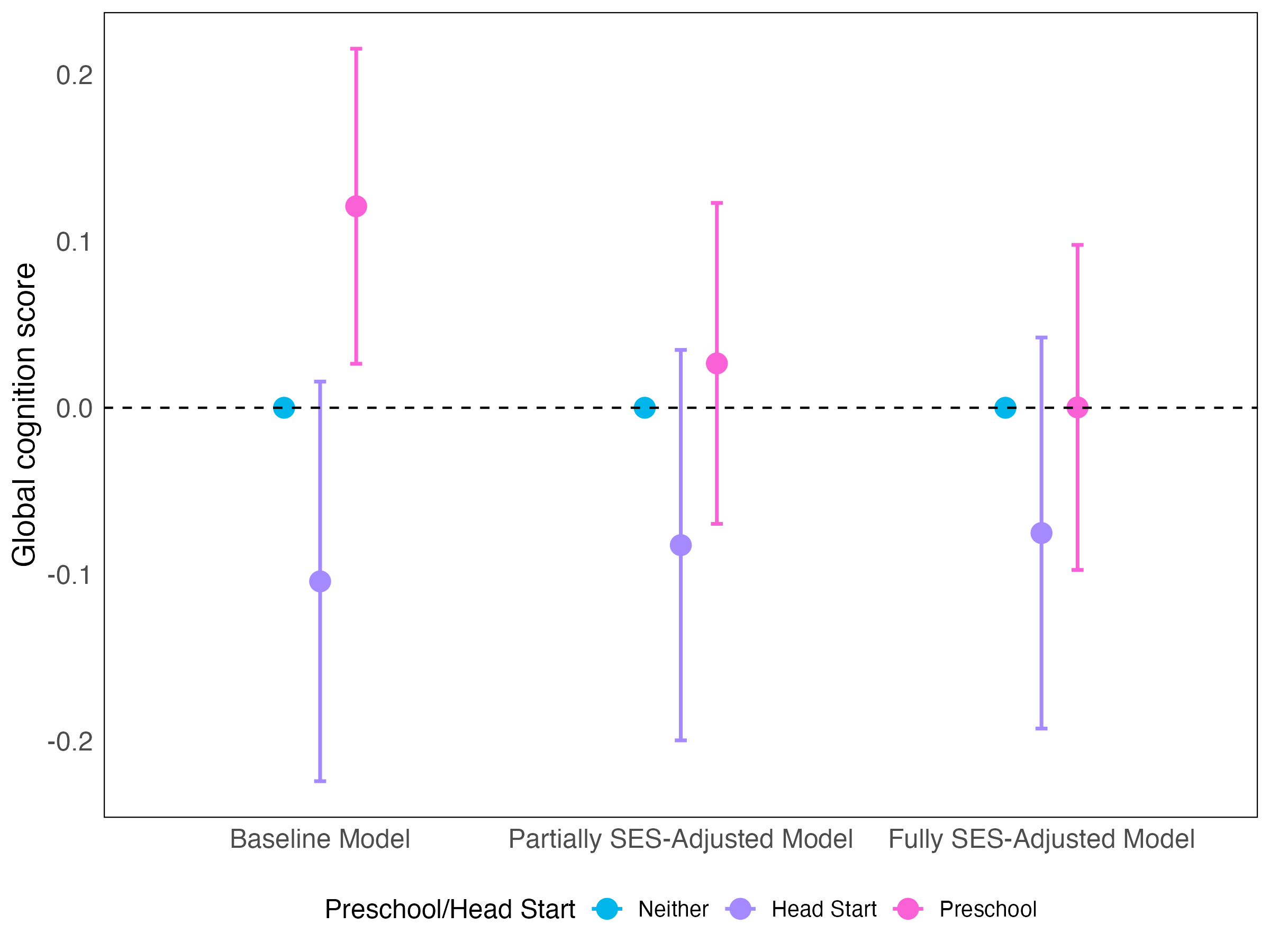
**

**
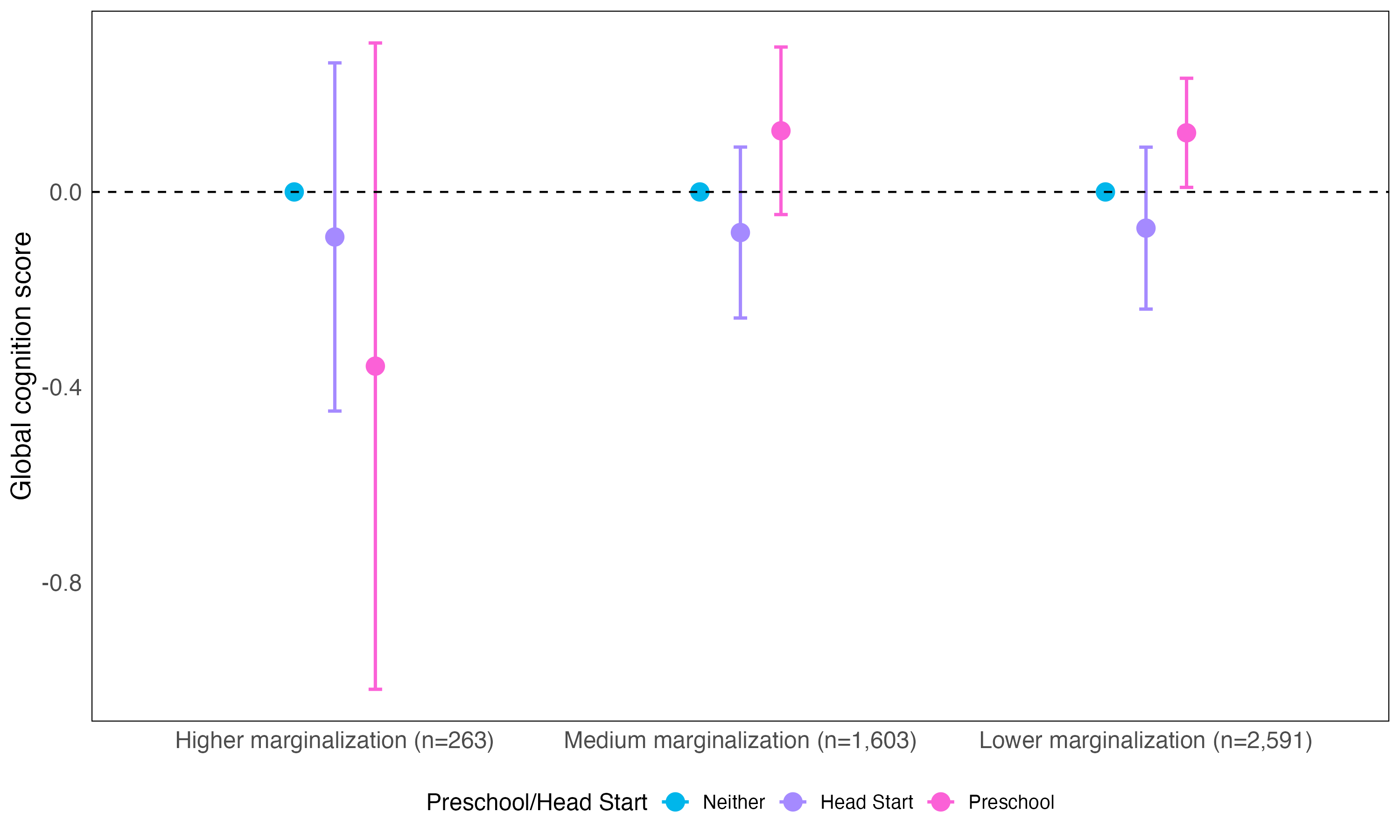
**

**
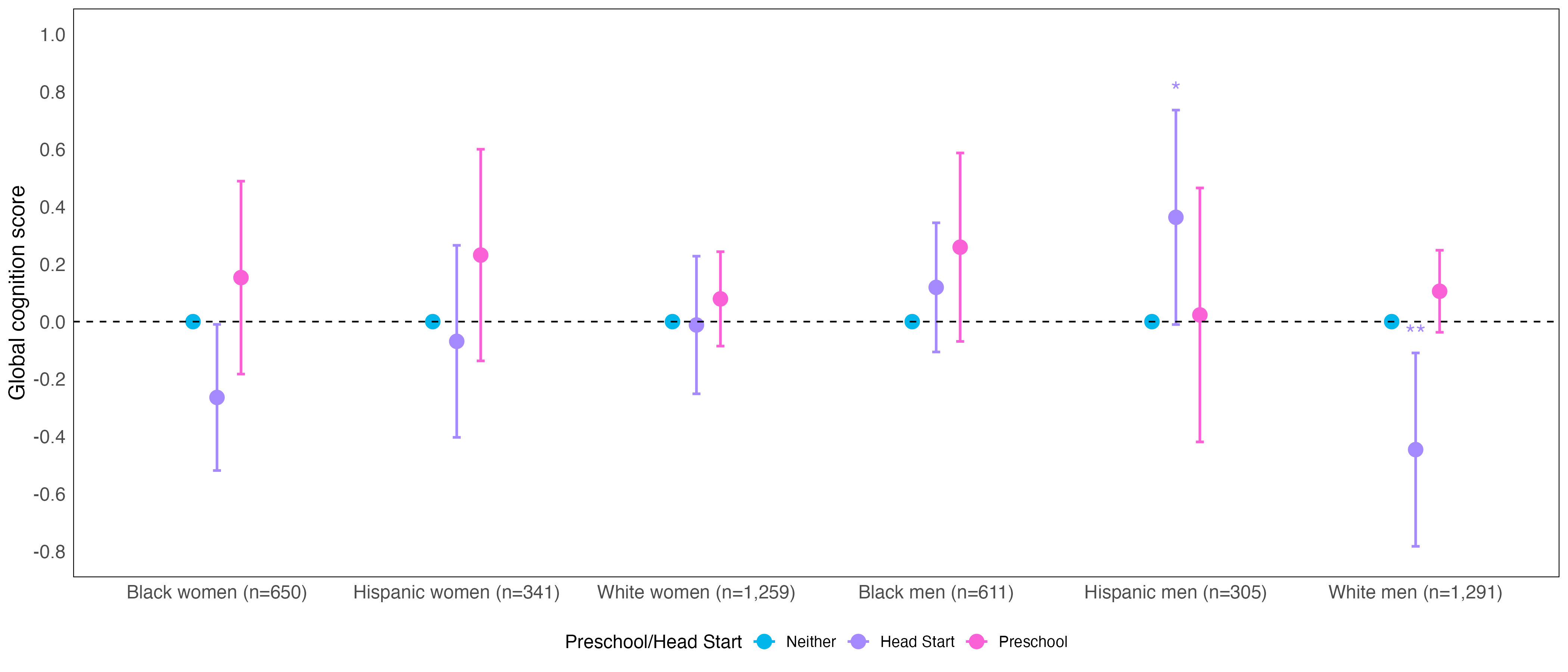
**

**eFigure 5.** Manuscript figures, using complete case analysis

Notes: Data drawn from the National Longitudinal Survey of Youth 1979. Complete case N=4,457.

Panel A. Association between exposure to early childhood education and midlife global cognition

Baseline Model adjusted for year of birth, race and ethnicity, sex, birth in a southern state, and mother’s and father’s nativity. Partially SES-Adjusted Model additionally adjusted for mother’s and father’s education. Fully SES-Adjusted Model additionally adjusted for rural residence, family poverty in the prior year, and mother’s and father’s occupation.

Panel B**.** Association between exposure to early childhood education and midlife global cognition, by index of family socioeconomic status marginalization

Models adjusted for year of birth, race and ethnicity, sex, birth in a southern state, and mother’s and father’s nativity. Coefficients were obtained using rotating reference groups.

Panel C**.** Association between exposure to early childhood education and midlife global cognition, by sex, race, and ethnicity

Asterisks denote statistical significance for the interaction terms, which represent the additional difference in midlife cognition for Head Start (vs no ECE) or preschool (vs no ECE) for the given subgroup compared to the reference group of White women. *Interaction term <0.1.

Models adjusted for year of birth, birth in a southern state, and mother’s and father’s nativity. Coefficients were obtained using rotating reference groups. NLSY created a category for “Non-Black, Non-Hispanic”, which we have labelled “Non-Hispanic White” based on prior research.


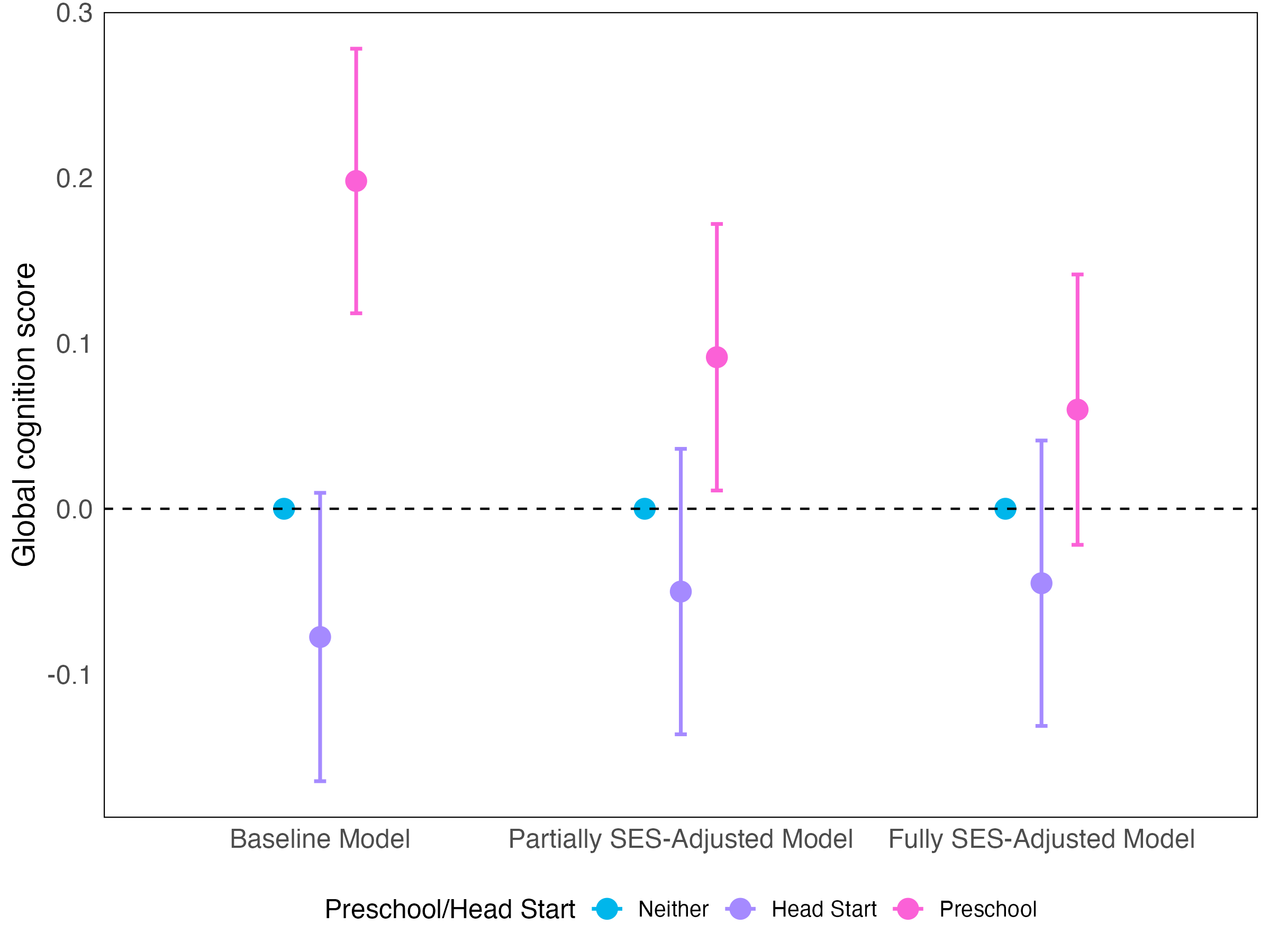


**
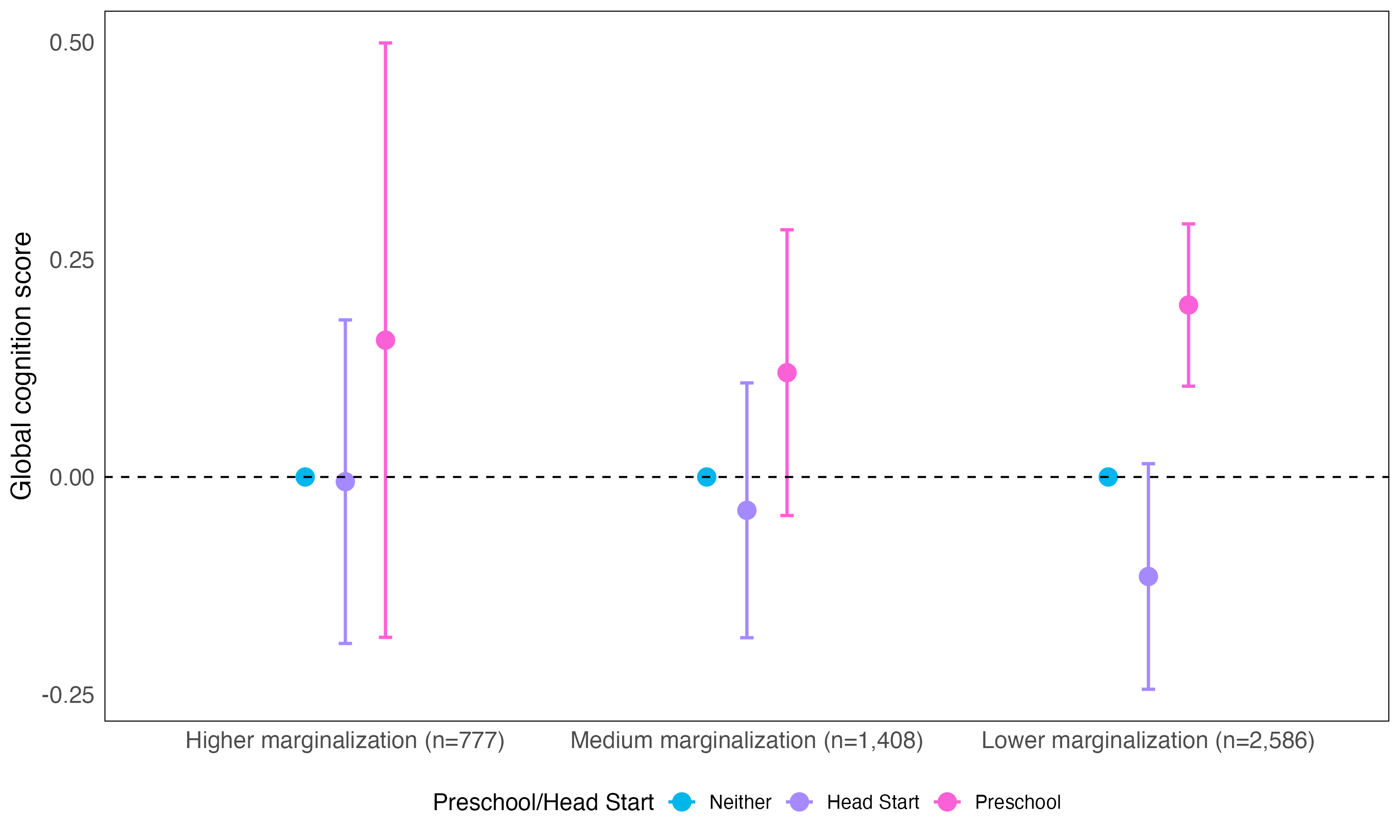
**

**
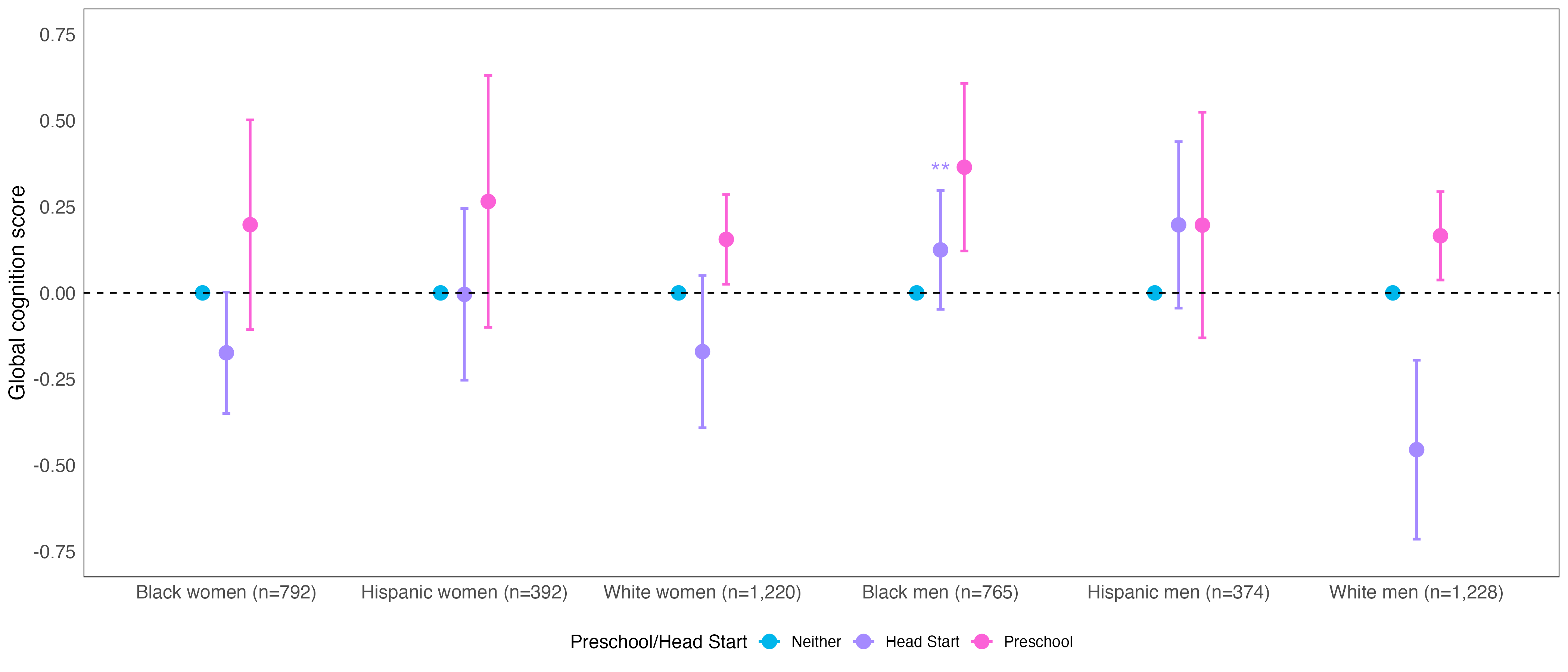
**

**eFigure 6.** Manuscript figures, excluding people born before 1960

Notes: Data drawn from the National Longitudinal Survey of Youth 1979. N=4,771.

Panel A. Association between exposure to early childhood education and midlife global cognition

Baseline Model adjusted for year of birth, race and ethnicity, sex, birth in a southern state, and mother’s and father’s nativity. Partially SES-Adjusted Model additionally adjusted for mother’s and father’s education. Fully SES-Adjusted Model additionally adjusted for rural residence, family poverty in the prior year, mother’s and father’s occupation, and mother’s and father’s presence in the household. NLSY created a category for “Non-Black, Non-Hispanic”, which we have labelled “Non-Hispanic White” based on prior research.

Panel B.Association between exposure to early childhood education and midlife global cognition, by index of family socioeconomic status marginalization

Models adjusted for year of birth, race and ethnicity, sex, birth in a southern state, and mother’s and father’s nativity. Coefficients were obtained using rotating reference groups.

Panel C. Association between exposure to early childhood education and midlife global cognition, by sex, race, and ethnicity

Asterisks denote statistical significance for the interaction terms, which represent the additional difference in midlife cognition for Head Start (vs no ECE) or preschool (vs no ECE) for the given subgroup compared to the reference group of White women. *Interaction term <0.1. **Interaction term <0.05.

Models adjusted for year of birth, birth in a southern state, and mother’s and father’s nativity. Coefficients were obtained using rotating reference groups.

**eTable 1.** Sample characteristics by early childhood education exposure, showing missingness on each variable

| **Variable** | **No Preschool/Head Start**, N = 4962^1^ | **Head Start**,  N = 1025^1^ | **Preschool**,  N = 1142^1^ |
| --- | --- | --- | --- |
| **Year of birth** | 1960 ± 2.2 | 1962 ± 1.4 | 1960 ± 2.2 |
| **Sex** |  |  |  |
| Male | 49% | 47% | 51% |
| Female | 51% | 53% | 49% |
| **Race and ethnicity** |  |  |  |
| Non-Hispanic White^2^ | 59% | 16% | 54% |
| Non-Hispanic Black | 25% | 69% | 31% |
| Hispanic | 16% | 15% | 15% |
| **Born in the South** |  |  |  |
| Non-south | 63% | 40% | 53% |
| South | 35% | 58% | 45% |
| Missing | 2.1% | 1.6% | 1.8% |
| **Father born in US** |  |  |  |
| In the US | 93% | 91% | 93% |
| Other country | 4.9% | 3.5% | 4.7% |
| Missing | 2.0% | 5.5% | 2.1% |
| **Mother born in US** |  |  |  |
| In the US | 95% | 96% | 94% |
| Other country | 5.0% | 3.8% | 6.0% |
| Missing | 0.3% | 0.2% | 0.2% |
| **Poverty prior year** |  |  |  |
| Not in poverty | 64% | 49% | 66% |
| In poverty | 17% | 34% | 15% |
| Missing | 19% | 17% | 19% |
| **Rural residence** |  |  |  |
| In town or city | 77% | 80% | 85% |
| In country or farm | 23% | 19% | 15% |
| Missing | 0.3% | 0.7% | <0.1% |
| **Father’s education** |  |  |  |
| < 8th grade | 13% | 17% | 9.9% |
| 8th grade to < High School | 23% | 27% | 18% |
| Highschool | 31% | 24% | 27% |
| > Highschool | 19% | 8.7% | 34% |
| Missing | 14% | 24% | 12% |
| **Mom education** |  |  |  |
| < 8th grade | 9.9% | 15% | 8.3% |
| 8th grade to < High School | 29% | 40% | 20% |
| Highschool | 40% | 30% | 36% |
| > Highschool | 14% | 8.9% | 31% |
| Missing | 6.6% | 6.2% | 4.6% |
| **Father’s occupation** |  |  |  |
| Employed, unskilled | 53% | 47% | 36% |
| Employed, skilled | 19% | 5.5% | 34% |
| Unemployed | 6.2% | 8.8% | 5.3% |
| Missing | 22% | 39% | 25% |
| **Mother’s occupation** |  |  |  |
| Employed, unskilled | 40% | 46% | 36% |
| Employed, skilled | 9.0% | 6.6% | 19% |
| Unemployed | 46% | 42% | 41% |
| Missing | 4.7% | 5.0% | 3.8% |
| **Father not in household/not known** |  |  |  |
| Dad in household | 83% | 66% | 80% |
| Dad not in household or never known | 17% | 34% | 20% |
| Missing | 0.2% | 0% | <0.1% |
| **Mother not in household/not known** |  |  |  |
| Mom in household | 98% | 98% | 99% |
| Mom not in household or never known | 1.7% | 1.9% | 0.8% |
| Missing | 0.2% | 0% | <0.1% |
| **Global cognition score** | 0.02 ± 0.98 | -0.22 ± 1.07 | 0.16 ± 0.96 |
| Missing N (%) | 2142 (43%) | 359 (35%) | 509 (45%) |
| **Memory subdomain score** | 0.00 ± 0.98 | -0.13 ± 1.04 | 0.15 ± 1.01 |
| Missing N (%) | 1718 (35%) | 212 (21%) | 414 (36%) |
| **Attention subdomain score** | 0.04 ± 0.97 | -0.28 ± 1.15 | 0.08 ± 0.95 |
| Missing N (%) | 764 (15%) | 215 (21%) | 175 (15%) |

^1^ %, n, or Mean ± SD

^2^ NLSY created a category for “Non-Black, Non-Hispanic”, which we have labelled “Non-Hispanic White” based on prior research

Notes: Data drawn from the National Longitudinal Survey of Youth 1979. N=7,129.

**eTable 2.** Detailed results: Association between exposure to early childhood education and midlife global cognition

| **Variable** | **Baseline Model** | **Partially SES-Adjusted Model** | **Fully SES-Adjusted Model** |
| --- | --- | --- | --- |
| **Early childhood education** | | | |
| No ECE | Ref | Ref | Ref |
| Head Start | -0.05 (-0.13, 0.04) | -0.03 (-0.11, 0.05) | -0.02 (-0.10, 0.06) |
| Preschool | 0.16 (0.09, 0.22) | 0.06 (-0.01, 0.12) | 0.03 (-0.03, 0.10) |

Notes: Data drawn from the National Longitudinal Survey of Youth 1979. N=7,129. Baseline Model adjusted for year of birth, race and ethnicity, sex, birth in a southern state, and mother’s and father’s nativity. Partially SES-Adjusted Model additionally adjusted for mother’s and father’s education. Fully SES-Adjusted Model additionally adjusted for rural residence, family poverty in the prior year, mother’s and father’s occupation, and mother’s and father’s presence in the household.

**eTable 3.** Detailed results: Association between exposure to early childhood education and midlife global cognition, by index of family socioeconomic status marginalization

| **Variable** | **Higher**  **marginalization (n=1,058)** | **Medium marginalization (n=2,188)** | **Lower**  **marginalization (n=3,883)** |
| --- | --- | --- | --- |
| **Early childhood education** | | | |
| No ECE | Ref | Ref | Ref |
| Head Start | 0.09 (-0.08, 0.27) | -0.03 (-0.17, 0.11) | -0.09 (-0.22, 0.04) |
| Preschool | 0.26 (0.01, 0.51) | 0.10 (-0.03, 0.23) | 0.14 (0.06, 0.22) |

Notes: Data drawn from the National Longitudinal Survey of Youth 1979. N=7,129. Models adjusted for year of birth, race and ethnicity, sex, birth in a southern state, and mother’s and father’s nativity. Coefficients were obtained using rotating reference groups.

**eTable 4.** Detailed results: Association between exposure to early childhood education and midlife global cognition, by sex, race, and ethnicity

| **Variable** | **Black women (n=1,179)** | **Hispanic women (n=569)** | **White women^1^ (n=1,890)** | **Black men (n=1,128)** | **Hispanic men (n=539)** | **White men^1^ (n=1,824)** |
| --- | --- | --- | --- | --- | --- | --- |
| **Early childhood education** | | | | | | |
| No ECE | Ref | Ref | Ref |  |  | Ref |
| Head Start | -0.09 (-0.25, 0.07) | -0.02 (-0.26, 0.22) | -0.18 (-0.39, 0.04) | 0.13 (-0.02, 0.29) | 0.14 (-0.09, 0.36) | -0.46 (-0.72, -0.21) |
| Preschool | 0.19 (-0.01, 0.40) | 0.14 (-0.13, 0.40) | 0.17 (0.06, 0.28) | 0.23 (0.05, 0.42) | 0.02 (-0.25, 0.29) | 0.14 (0.03, 0.25) |

^1^ NLSY created a category for “Non-Black, Non-Hispanic”, which we have labelled “Non-Hispanic White” based on prior research

Notes: Data drawn from the National Longitudinal Survey of Youth 1979. N=7,129. Models adjusted for year of birth, birth in a southern state, and mother’s and father’s nativity. Coefficients were obtained using rotating reference groups.

**eTable 5.** Detailed results: Association between exposure to early childhood education and midlife global cognition, with interaction term for index of family socioeconomic status marginalization

| **Variable** | **Coefficient** | **95% CI** | **p-value** |
| --- | --- | --- | --- |
| **Early childhood education** |  |  |  |
| No ECE | Ref | - | - |
| Head Start | -0.09 | (-0.22, 0.04) | 0.16 |
| Preschool | 0.14 | (0.06, 0.22) | <0.01 |
| **Family SES** |  |  |  |
| Lower marginalization | Ref | - | - |
| Medium marginalization | -0.21 | (-0.28, -0.14) | <0.01 |
| Higher marginalization | -0.51 | (-0.62, -0.40) | <0.01 |
| **ECE * Family SES interaction** |  |  |  |
| Head Start * Lower marginalization | Ref | - | - |
| Preschool * Lower marginalization | Ref | - | - |
| Head Start * Medium marginalization | 0.06 | (-0.13, 0.24) | 0.55 |
| Preschool * Medium marginalization | -0.04 | (-0.19, 0.11) | 0.60 |
| Head Start * Higher marginalization | 0.19 | (-0.03, 0.40) | 0.09 |
| Preschool * Higher marginalization | 0.12 | (-0.14, 0.38) | 0.37 |
| **Interaction test: ECE * Family SES** |  |  |  |
| Likelihood ratio test statistic | Statistic=1.15 | - | 0.33 |

Notes: Data drawn from the National Longitudinal Survey of Youth 1979. N=7,129. Models adjusted for year of birth, race and ethnicity, sex, birth in a southern state, and mother’s and father’s nativity. Coefficients were obtained using rotating reference groups.

**eTable 6.** Detailed results: Association between exposure to early childhood education and midlife global cognition, with interaction term for sex, race, and ethnicity

| **Variable** | **Coefficient** | **95% CI** | **p-value** |
| --- | --- | --- | --- |
| **Early childhood education** |  |  |  |
| No ECE | Ref | - | - |
| Head Start | -0.18 | (-0.39, 0.04) | 0.11 |
| Preschool | 0.17 | (0.06, 0.28) | <0.01 |
| **Sex, race, and ethnicity** |  |  |  |
| Non-Hispanic White women^1^ | Ref | - | - |
| Non-Hispanic White men^1^ | -0.15 | (-0.22, -0.08) | <0.01 |
| Black women | -0.50 | (-0.61, -0.40) | <0.01 |
| Black men | -0.77 | (-0.88, -0.67) | <0.01 |
| Hispanic women | -0.44 | (-0.55, -0.32) | <0.01 |
| Hispanic men | -0.60 | (-0.72, -0.48) | <0.01 |
| **ECE * Sex/race/ethnicity interaction** |  |  |  |
| Head Start * White women | Ref | - | - |
| Preschool * White women | Ref | - | - |
| Head Start * White men | -0.29 | (-0.62, 0.05) | 0.09 |
| Preschool * White men | -0.03 | (-0.18, 0.12) | 0.72 |
| Head Start * Black women | 0.08 | (-0.18, 0.35) | 0.53 |
| Preschool * Black women | 0.03 | (-0.20, 0.25) | 0.83 |
| Head Start * Black men | 0.31 | (0.04, 0.57) | 0.02 |
| Preschool * Black men | 0.06 | (-0.15, 0.27) | 0.56 |
| Head Start * Hispanic women | 0.15 | (-0.17, 0.48) | 0.35 |
| Preschool * Hispanic women | -0.03 | (-0.32, 0.25) | 0.82 |
| Head Start * Hispanic men | 0.31 | (0.00, 0.62) | 0.05 |
| Preschool * Hispanic men | -0.15 | (-0.44, 0.14) | 0.31 |
| **Interaction test: ECE * Sex/race/ethnicity** | | | |
| Likelihood ratio test statistic | Statistic=2.51 | - | 0.01 |

^1^ NLSY created a category for “Non-Black, Non-Hispanic”, which we have labelled “Non-Hispanic White” based on prior research

Notes: Data drawn from the National Longitudinal Survey of Youth 1979. N=7,129. Models adjusted for year of birth, birth in a southern state, and mother’s and father’s nativity. Coefficients were obtained using rotating reference groups.
